# Piecewise Polynomial Representations of Genomic Tracks

**DOI:** 10.1371/journal.pone.0048941

**Published:** 2012-11-15

**Authors:** Maxime Tarabichi, Vincent Detours, Tomasz Konopka

**Affiliations:** 1 IRIBHM, Université Libre de Bruxelles, Brussels, Belgium; 2 Welbio, Université Libre de Bruxelles, Brussels, Belgium; Auburn University, United States of America

## Abstract

Genomic data from micro-array and sequencing projects consist of associations of measured values to chromosomal coordinates. These associations can be thought of as functions in one dimension and can thus be stored, analyzed, and interpreted as piecewise-polynomial curves. We present a general framework for building piecewise polynomial representations of genome-scale signals and illustrate some of its applications via examples. We show that piecewise constant segmentation, a typical step in copy-number analyses, can be carried out within this framework for both array and (DNA) sequencing data offering advantages over existing methods in each case. Higher-order polynomial curves can be used, for example, to detect trends and/or discontinuities in transcription levels from RNA-seq data. We give a concrete application of piecewise linear functions to diagnose and quantify alignment quality at exon borders (splice sites). Our software (source and object code) for building piecewise polynomial models is available at http://sourceforge.net/projects/locsmoc/.

## Introduction

Genomic studies often involve associations of measurements with genomic coordinates. In high-throughput sequencing studies, for example, one has information about genomic features such as copy-number, expression, or binding affinity at near single-nucleotide resolution. Such tracks can be thought of describing functions in a one-dimensional space parameterized by a natural coordinate. We here discuss the role piecewise polynomial curves (PPCs) can play to conveniently store, process, and interpret such data. We argue PPCs can be incorporated into existing analysis pipelines and open opportunities to view data from new perspectives.

PPCs are functions over a one-dimensional space that can be formulated as simple polynomials within contiguous finite intervals. Piecewise constant functions, which are special instances of PPCs, describe properties that are fixed on a genomic range and indicate the positions where these properties change discontinuously. They are already in widespread use in genomics under the guise of run-length encodings or segmentations, for example in context of studying regions of copy-number variation (see [1]–[3] and references therein).

**Figure 1:**
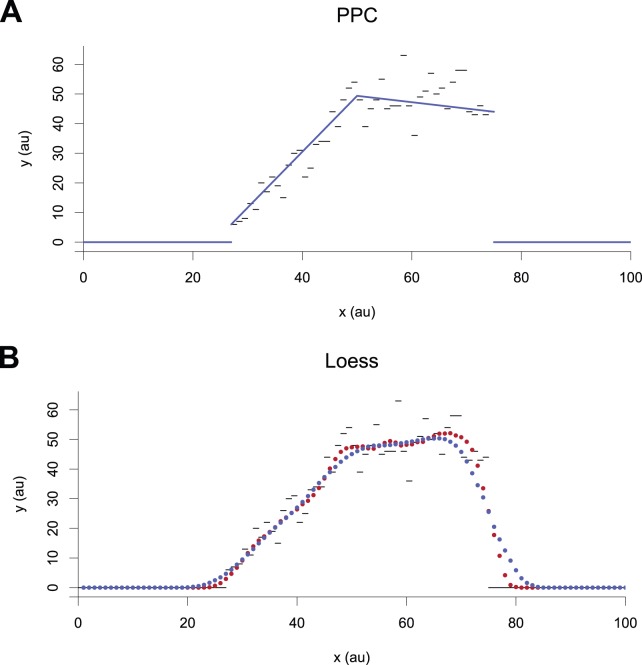
Modeling of signals. A sample signal (gray bars) modeled/smoothed using (A) a piecewise-linear function (blue line) and (B) loess smoothing (blue and red dots, different settings). The PPC representation captures trends and highlights discontinuities.

Higher-order PPCs, i.e. piecewise linear or piecewise quadratic functions, are more general objects capable of describing both abrupt and gradual changes. Similarly to the discontinuities already mentioned, gradual changes can also be informative. For example, GC content at a locus is actually a property of a neighborhood and thus changes slowly from one position to the next. In high-throughput sequencing studies, signals have short range correlations associated with the read length. Rolling patterns can be observed in alignment coverage as well as in derived tracks such as high-resolution mappability scores [4]. In such applications, it is desirable to optimally encode a signal and piecewise-linear segments are natural candidates for the job (Figure 1).

The motivation for replacing raw data with functional models is similar to that for using regression to summarize linearly related points. Good models capture the essence of data, suppress noise, and provide an output that can be interpreted meaningfully. In this sense modeling can be regarded as a lossy compression scheme. Whereas it can be dangerous if applied too aggressively, it need not be detrimental to downstream analysis. For instance, in another context, lossy compression of base quality scores in fastq files has been shown not to be detrimental to identification of single-nucleotide variants [5], [6]. At the same time, the functional form of the PPCs can enable novel types of analysis. In the literature on modeling of gene-expression time-series, for example, the ability to describe data in terms of mathematical functions is a key ingredient in algorithms inferring regulatory mechanisms between genes [7], [8]. In genomics, the functional form can describe a region as opposed to individual locations and thereby offers a robust framework to compare trends and biases between technologies and samples.

In practice, building effective piecewise polynomial models for genomic signals (Figure 1) is nontrivial for several reasons. First, the length of a chromosome or genome, denoted as 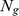, can be on the order of 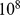 or more. Second, genomic signals often contain a large number of domains with distinct patterns. Characterizing these events is often the objective of bioinformatic studies, for example in finding copy-number breakpoints or splice-sites in transcriptomics, but the space of possible event locations scales super-exponentially with 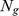. Third, although genomic features are sometimes measured at near single-nucleotide resolution, relevant signals often show regional correlation. These can reflect a certain read length in a high-throughput sequencing study or reveal biological properties such as the distribution of genes along a genome. Whatever the reason, regional correlations imply that a signal of actual length 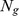 can often be encoded using much fewer values via run-length encoding. Denoting 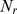 as the number of runs, one can have 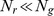. Thus, run-length encoding provides a natural reference to which curve smoothing can be compared to.

In light of these characteristics, one can understand why polynomials have been used in internal processing in some applications involving short signals (e.g. [9]), but they have rarely been used for representation of long genomic tracks: Conventional data smoothing methods turn out to be inappropriate for genomics due to computational complexity or due to their smoothing assumptions. Methods developed for short signals are impractical if they require 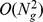 time or space resources. This applies to many sophisticated segmentation methods which work top-down [9]. Methods which work in the spirit of a convolution, for example a basic moving average or locally weighted regression (loess) can require only 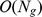 or 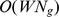 resources, where 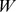 is the width of a predefined window. However, they require the user to specify a window width, which may not be obvious in practice [2]. Another disadvantage of convolution methods is that they blur sharp contrast (Figure 1). This is undesirable for biological reasons and also leads these methods to provide output that is less amenable to run-length encoding than the original signal. Thus, although these smoothings eliminate local noise, they can inflate rather than compress the size of a signal.

In order to smooth signals in regions where it is appropriate and simultaneously preserve discontinuous boundaries, an adaptive approach employing functional representations is required. Segmented regression [10] and some spline-based techniques [11], [12] fall in this category. Some implementations use greedy search and/or invoke constraints limiting the model complexity, but top-down techniques can still require quadratic resources in time or memory when the signal contains a large number of domains. Implementations from non-genomic fields typically also assume maximal continuity conditions. This does not immediately rule out their use in genomics, but imposes unnecessary conditions. Lastly, whereas some implementations rely on minimizations of objective functions, a more intuitive approach for pattern detection in genomics is based on distributions (poisson, binomial, generalized t-distributions, etc.).

We here describe a general tool appropriate for producing PPCs for genomic signals. The algorithm builds on selected techniques from the spline literature and adapts them to the genomic context. As a result, it is suitable for processing very long signals with multiple domains in time sub-quadratic in the length of the input. Technically, the algorithm does not attempt to define and minimize a global objective function for a piecewise polynomial model. Instead, it produces approximate or acceptable models that satisfy a-priori set conditions and show discontinuities where these conditions are broken (Figure 1). It accepts run-length encoded curves as input and thereby exploits one of the regularities of genomic sequences. It can output both continuous and discontinuous models.

The rest of this paper is structured as follows. In the results section, we illustrate some of the possible applications of piecewise polynomial curves in bioinformatics with examples: signal compression, improved detection of genomic copy-number from array and sequencing data, and characterization of alignment artifacts and gene-expression patterns in RNA-seq. Many of these are supplemented by additional calculations in the Text S1. In the discussion section, we summarize the applications and comment on the general outlook. Our algorithm for generating PPC models is reviewed in the methods section and in the Text S1.

## Results

In this section, we illustrate the versatility of piecewise polynomial curves in bioinformatic applications through a series of examples.

### Signal Compression

To demonstrate the use of PPCs as a compression scheme, we generated synthetic sequencing data by placing single reads of length 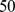 nt uniformly at random on ‘chromosomes’ of length 1Mnt. We created data of various coverage depth and saved the coverage tracks using run length encoding. We then calculated PPCs models with poisson error model for all the signals (Methods). As the smoothing algorithm is stochastic and produces different solutions when started with different seeds, we repeated the smoothing procedure 10 times for each data signal. We measured memory requirements to represent the coverage tracks and their PPC models by loading the signals into tables in R and applying the object.size function. Results show that high-coverage signals can be compressed with high inverse compression ratios (Table 1 and Text S1 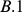). Low coverage signals are ‘compressed’ less as knot operations pass the acceptance criteria less frequently (Methods) and because their run-length encoding is more efficient to begin with.

**Table 1:** Compression due to PPCs.

		Memory (ICR)		Mean Len.	
P	O	8r/nt	250r/nt	8r/nt	250r/nt
1	0	3.6	22.1	21.3	39.0
1	1	2.2	13.1	22.1	38.2
2	0	8.4	67.8	51.0	125.9
2	1	4.9	30.2	48.8	90.1

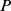
 and 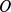 denote error model parameter 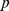 and the order of the PPCs, respectively. ICR stands for inverse compression ratio. Mean Len. describes the typical segment length in the PPCs. For reference, mean lengths in uncompressed signals are 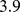 and 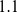 for the low and high coverage density signals. r/nt stands for reads per nucleotide. Note: since the PPC algorithm is stochastic, values may vary from signal to signal and from one run to another.

**Figure 2:**
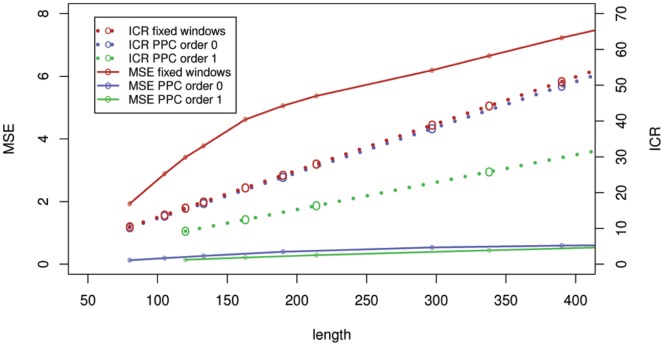
Representations of mappability track of human chromosome 21. MSE is mean square error between a model and the original track (solid lines, left scale). ICR is inverse compression ratio (dotted lines, right scale). Red points summarize performance of an approach averaging the track in windows of fixed width. Blue and green points summarize performance of PPCs with order 0 and 1, respectively. Points represent computed values, lines are drawn to show trend. The plot shows that for given ICR, the PPC models produce fits with lower mean square error than averages in fixed windows.

Similar compression performance is achievable on real signals originating from sequencing projects. We downloaded human genome mappability tracks for 100bp paired-end reads [4] and saved them as run-length-encoded signals. We then computed PPC models using various settings (Figure 2). Constraining first order PPC to give a model with 1% maximal deviation, we found the tracks can be represented with inverse compression ratios between 5 and 10 (depending on chromosome). Allowing deviations of 2% can push the ratios up to 20.

### Copy Number Detection

Genomic regions of constant copy number are often described via long segments [1]–[3]. As these are actually piecewise polynomial curves of order zero, we here discuss the application of our smoothing algorithm to that context. The aim here is not to propose a new copy-number calling procedure, but rather to point out certain issues where the PPC smoothing may be of use.

#### Array-based signals

In an array experiment studying copy-numbers, DNA from a sample is hybridized on probes sampling positions along a genome. A well-regarded algorithm [1] for copy-detection using these probes is circular binary segmentation (CBS) [13]. It begins by treating a chromosome as a single segment and then breaks it up iteratively if co-localized groups of probes have, according to an empirical t-test, statistically different mean intensity from the rest. Our algorithm also supports segmentation using the t-test (Methods), but works differently than CBS in that it starts by treating single probes as segments and then joins them together.

**Figure 3:**
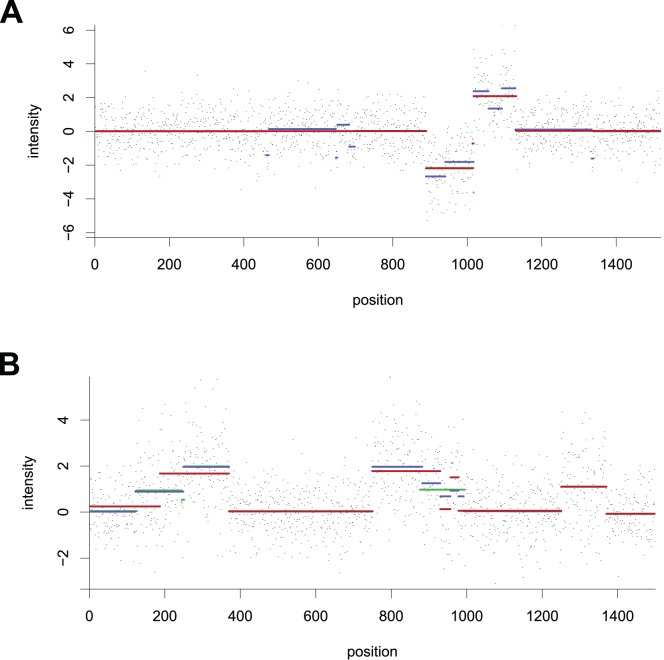
CGH signals with aberrations. Dots are probe intensities, horizontal lines indicate true copy-number levels (green), and segments output by CBS (red) and the median of 5 PPC runs (blue). Where some lines are not visible, it is because multiple segments overlap. Both signals contain 1500 probes. (A) Situation where CBS outperforms the PPC method. (B) Situation with multiple copy-number events where PPC segments outperform CBS (missed events around positions 200 and 950).

To compare these closely related algorithms, we started by simulating synthetic signals of lengths 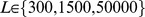 probes (1250 signals for each length) by sampling random numbers from a normal distribution with zero mean and unit standard deviation. We then added between one and ten events in windows of length in the range 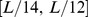 and amplitudes selected from the same normal distribution (Figure 3). We ran CBS (default parameters) and our algorithm with the t-test method (Methods, 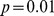) on the simulated data. Since the PPC algorithm is stochastic, we also considered composite PPC segmentations by taking median segment values from 5 independent runs. For each chromosome, we computed the probe-wise sum of squared distances between segmented and simulated signals. Finally, we measured the proportion of times CBS gave a lower error than PPC (Table 2). This gave us a measure of segmentation quality independent of copy-number calling.

**Table 2:** Array segmentation methods.

		Number of CN events			
Passes	Probes	1	3	6	9
1	300	0.63	**0.48**	0.51	**0.37**
1	1500	0.94	0.88	0.75	0.67
1	50000	1	1	1	1
5	300	0.55	**0.38**	**0.34**	**0.25**
5	1500	0.90	0.78	0.57	**0.41**
5	50000	1	1	1	1

Entries in the table indicate the proportion of times (out of 125 observations in each category) that top-down segmentation outperforms the bottom-up approach. Values smaller than 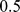 (highlighted) indicate the bottom-up approach (PPC) is more appropriate than top-down (CBS) for short signals and signals with many copy-number events. Performance improves by averaging several PPC models. Note: since the PPC algorithm is stochastic, proportions can vary slightly from one run to another (variation is on the order of 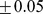).

Results show CBS often outperforms our algorithm for signals with few copy-number events (Figure 3A) and for signals with very large number of probes in which each event is supported by many probes. In these situations, the error attributed to the PPC approach can be high. However, it originates from very short segments (Figure 3A), which can have minimal impact on copy-number calling (Text S1 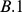). For signals with coarse to medium resolution and a large (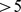) number of events, which are not uncommon when studying cancer samples, it is the bottom-up approach that often gives smaller errors (Figure 3B). We therefore conclude that CBS is an excellent tool in most situations, but that the PPC approach may be an interesting alternative in niche analyses.

Text S1 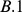 contains additional calculations relating to array segmentation, including comparisons with two other segmentation methods, GLAD [14] and HMM [15].

#### Sequencing-based signals

In the context of sequencing, one approach to detect copy numbers changes is through read depth [2] (alternative methods involve analysis of insert sizes between paired-end reads). The underlying logic is that a genomic region that has been deleted will not be represented in a sample. Thus, when sequenced reads are aligned to a reference genome, the coverage depth within that region will be reduced relative to other regions. Similar reasoning applies when copy number is increased.

Because coverage signals in sequencing samples have a finite auto-correlation length, many algorithms for copy-number detection developed for microarray platforms tend to produce segmentations with too many events, taking a prohibitive amount of time in the process [2], [16]. A workaround, used in all methods for segmentation of sequencing coverage, is to divide a signal into non-overlapping windows of fixed width, compute coverage in each window, and then perform segmentation or copy-number calling using those values. This works because the averages in neighboring windows become independent and thus resemble data produced by sparse microarrays. The drawback of this approach, however, is that a long window width required to remove the auto-correlation implies that breakpoints cannot be inferred at resolution better than the window width. It follows that it may be beneficial to define short segments via an approach like PPC instead of adopting fixed intervals by *fiat*.

To investigate this issue, we again generated synthetic data. We first created a set of 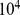 paired chromosomes with random sequence and considered them as a reference genome. We then produced a derived genome with modified copy numbers by selecting a subsequence of around 2 kilobases in each chromosome and deleted, duplicated, or left it in place in either one or both copies. Next, we generated perfectly matching single-end 75bp reads from this aberrant genome and obtained a sample with coverage depth 30. We aligned the reads to the original genome using Bowtie [17] and computed the coverage tracks.

**Figure 4:**
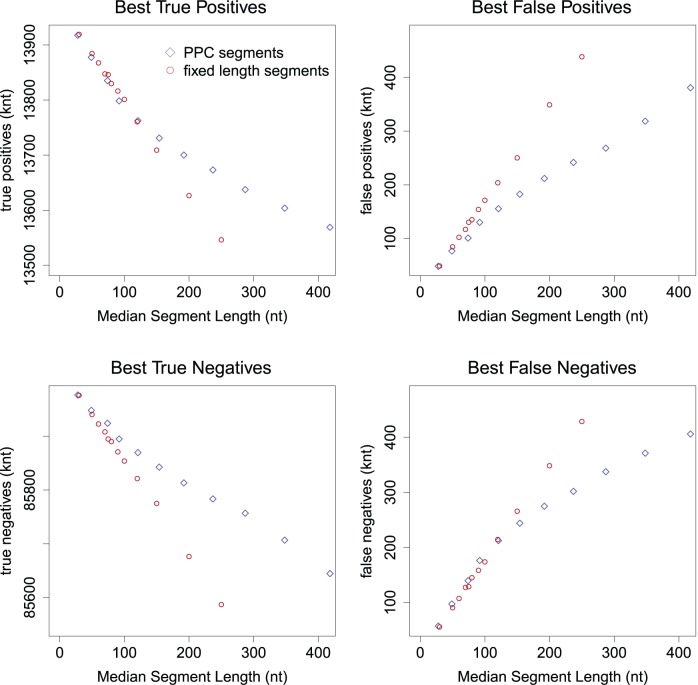
Fixed vs dynamic windows of sequencing coverage. Red circles indicate the best possible classification performance based on signal processed into fixed windows of various lengths. Blue diamonds show equivalent measures for dynamically defined segments with a certain median length. Note the scales on the vertical axes do not reach zero.

Next, we segmented the coverage signals using fixed windows of various lengths and using our approach with various parameters settings, which each led to segmentations with a characteristic (median) length. We labeled each window/segment as copy-number aberrant if more than half of its width overlapped a region with actual copy-number change. This procedure is somewhat arbitrary, but is blind to the segmentation method. We can thus regard these labels as the best possible classification achievable by a perfect copy-number caller given the segmentations. We computed the number of bases in the synthetic genome that can be regarded as true positive, false positive, true negative and false negatives (Figure 4). The results show that for segments with median length greater than the read length, the PPC segments consistently outperform the fixed-window approach (higher true positive and true negative rates, lower false positive and false negative rates). For shorter segments, the perfect classifiers would give comparable results with fixed windows and PPC segments.

The calculation based on the “perfect” classifier supports the argument that working with dynamically defined segments can improve performance relative to fixed-width windowing. However, the relevance of the calculation to actual practice relies on the assumption that real classifiers show similar trends. To see this is indeed the case, recall that segmentation algorithms based on long windows generally give acceptable results while windows that are much shorter than the auto-correlation length do not [2]. Thus, real classifiers can plausibly achieve close-to-perfect performance with long windows but not for short ones. To verify this intuition explicitly, we performed classification of the synthetic data using support vector machines. The details and results, consistent with the reasoning above, appear in Text S1 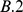.

To conclude this section, we discuss normalization of real sequencing data. Raw coverage signals are often normalized to remove biases due to read capture or mappability. To demonstrate that avoiding fixed-length windows can enhance this normalization step, we started by downloading alignments (hg18) of full-genome sequencing sample NA12891 from the 1000 Genomes Project [18]. We computed the coverage tracks and smoothed them (poisson smoothing, 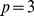) to obtain variable-length windows with median lengths around 100 and mean lengths around 150 (numbers vary by chromosome). We then looked up the fraction of each segment overlapping bases C or G and computed the mutual information (MI) between CG content and coverage depth. We found MIs around 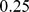 (Text S1 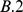). For each chromosome, we divided the coverage track into windows of fixed width equal to the median and mean found by the PPC approach and then recomputed the MIs. We found that in all but two chromosomes, calculations using dynamically defined segments produced higher mutual informations than the fixed window approach (differences are around 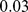). This result opens the opportunity to study CG bias in sequencing data by controlling for length of dynamical windows. One possibility for doing this would be to adapt the technique presented in [16].

### RNA-seq Coverage

In addition to enhancing segmentation analyses, the generality of the PPC modeling framework also permits us to explore genomic data in novel ways. We here explore PPCs of linear order in the context of RNA sequencing.

**Figure 5:**
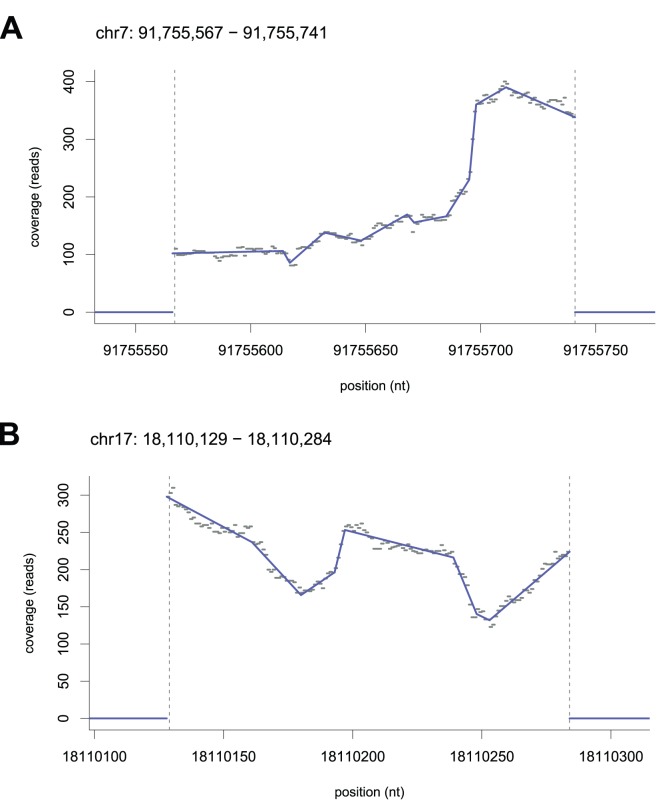
Smoothing of coverage tracks. Randomly selected exons with mean coverage density above 50, exon width greater than 100, and where segment slopes near both boundaries are (A) positive in inward direction and longer than 25nt; (B) negative in inward direction and longer than 25nt. Dashed lines indicate exon boundaries. Examples capture segments contributing to the tails of the histograms in Figure 6.

In an RNA sequencing study, reads originate from mRNA transcripts rather than raw DNA. Thus coverage is high predominantly on exons of annotated genes and low elsewhere. The coverage depth on the exons depends on gene expression level, splicing events, and can contain a number of biases (e.g. [19], [20]). As a result, coverage can vary even within single genes or even single exons (Figure 5).

We began by studying a coverage track from a human thyroid sample from the publicly available bodymap dataset (Illumina sequencing, 50bp paired-end reads; dataset consists of 16 samples originating from various human healthy tissue types) [21]. We aligned the sample on the hg19 reference genome using Tophat [22] (v. 2.0.0) with and without exon junction libraries, and computed the coverage tracks. We found genomic ranges labeled as exons in the refGene annotation set [23] and eliminated overlapping regions.

We computed piecewise linear models using poisson smoothing with parameter 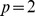 (Figure 5). As the resulting PPCs can be continuous or discontinuous, we looked at the positions of discontinuities. Among the exons with coverage density greater than the median, 87% showed a discontinuity at the position specified as a boundary by the refGene annotation or within 3 bases from it. The high rate is consistent with the notion that non-exonic sequence is spliced out and quickly degraded. However, the result opens the opportunity to identify and study the cases where an RNA-seq signal does not conform.

**Figure 6:**
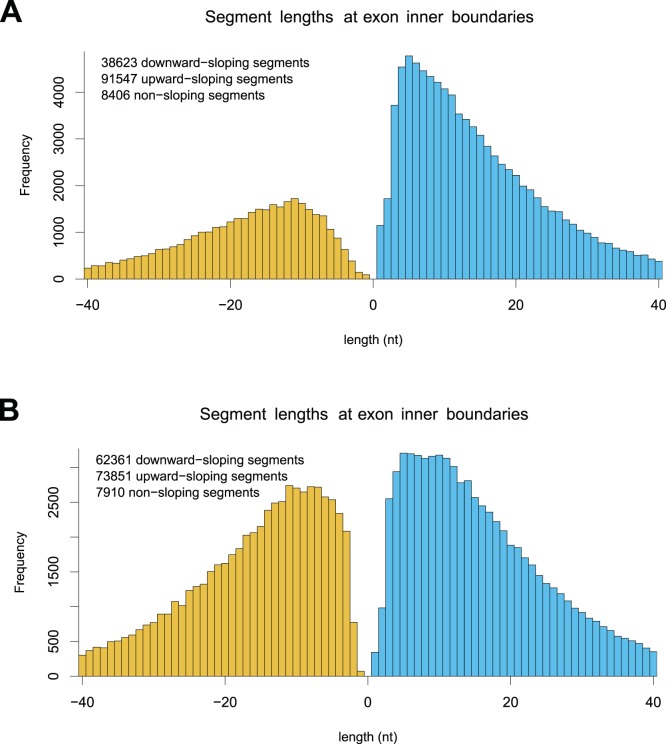
Segments close to inner exon boundaries Positive (negative) lengths indicate upward (downward) sloping segments. Colors emphasize sign of slope. (A) Unsupervised alignment. (B) Using splice junctions library.

We investigated slopes near boundaries between long (width 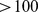 nt) and highly covered exons (depth 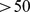) and neighboring introns and intergenic regions. Unsurprisingly, a vast majority of segments just outside exons are either flat or downward sloping as seen from the exon boundary. Segments on inside boundaries, in contrast, are more likely to be upward sloping than downward sloping as seen from the exon boundary (Figures 5 and 6, and Text S1). The bias is acceptable at the 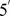 and 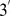 ends of genes and indeed also appears near boundaries in synthetically generated signals. However, given the accepted view that RNA transcripts are long strands spanning multiple exons, a strong bias is not expected in general.

Strong slope biases are qualitatively observed in other samples in the bodymap dataset, including those from other tissues and those sequenced with 75 bp and 100 bp single-end protocols. They are also present when the same computation is repeated counting only segments from inner exons, i.e. those not classified as first or last in any known gene isoform. The universality is therefore likely an artifact due to well-known difficulties in aligning relatively short reads across exon junctions. Indeed, it is greatly reduced when alignment is performed in supervised mode using the same splice junction library used in the slope bias test (Figure 6B). However, the bias is not removed completely (binomial test, 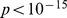).

As the histograms in Figure 6 contain information about the sign of the slopes but not their magnitudes, it is in principle possible that the bias arises due to segments that are very shallow. We thus investigated in more detail the subgroup of boundary segments on inner exons that are steep (slope magnitude greater than 0.7) and longer than 25nt. We counted 7086 upward- and 5871 downward-sloping segments, respectively (binomial test, 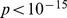), showing that the bias is not purely cosmetic.

Although the bias might not carry direct biological meaning, it does have important consequences. For instance, lower apparent coverage near exon boundaries reduces power to detect variants or to use those variants to estimate allelic ratios. This may not be a problem for highly-expressed genes in well-annotated organisms, but becomes increasingly relevant when studying weakly expressed genes, especially if they are not included in the splice library or when overall coverage is low. In addition, taking the slope bias as an indicator of alignment quality prompts questions about the reliability of the alignment near exon boundaries and possibly elsewhere.

To finish this section, we mention that steep and long segments also occur well away from exon boundaries. This may indicate additional artifacts or perhaps novel locations of transcription start/end sites. Previous reports [20] noticed sequence-specific biases in read start and end locations, so we computed position weight matrices (PWM) for sequences around features found by the PPCs. We observed strong signals in PWM computed around exon boundaries (as controls) but did not find similarly striking effects around steep long segments entirely inside exons and far from the boundaries. Thus, these coverage patterns are either due to sampling or are regulated in ways not captured by PWM. In any case, the ability to evaluate slopes at loci opens opportunity to systematically study trends across as well as within samples.

## Discussion

Piecewise polynomial curves (PPCs) are a compact and versatile description of one-dimensional signals that have different behaviors in different domains. Encoding genomic signals using PPCs can serve two main purposes: to compress data by eliminating noise, and to provide a representation that has greater interpretability value than the original.

We presented a general tool for constructing PPC models for genomic data. It is related to conventional model-building methods, but unlike regression or smoothing splines, the method does not aim to minimize a global objective function. Instead, the goal is to provide PPC representations of the input data that respects user-specified criteria. A positive consequence of this is that outliers in the data are never quietly incorporated into a model. Rather, they create breakpoints/discontinuities that can be later identified and interpreted as needed. This approach also allows us to take advantage of peculiarities of genomic data such as run-length encoding of the input.

The smoothing program provides output in 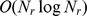 time, where 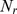 is proportional to the length of the input (measured in bytes, which can be smaller that length of signal due to run length encoding). In contrast to smoothing methods such as loess, the PPC representation can sometimes provide significant compression both in memory and disk. Moreover, PPCs can be stored in a tabular data-structure similar to those currently used for piecewise-constant curves (e.g. wiggle files). They thus provide an accessible extension to existing data storage formats.

Extensions to the described method are possible, particularly in the direction of applying Monte-Carlo techniques (e.g. [11], [12]). Our algorithm could provide an input to such an approach. We did not investigate such an extension here as it would increase running time.

We demonstrated piecewise constant curves can be useful in identifying copy-number changes in both array- and sequencing-based data. The bottom-up approach is particularly suited for studying signals with multiple copy-number changes. For sequencing data, the dynamic formation of segments is an alternative to windowing with a-priori specified width, which is an essential stage in all currently available methods. Furthermore, the generality of the PPC could perhaps be exploited in the future to build even better classifiers. For example, information from piecewise-linear representations of the coverage signal may help improve breakpoint detection at the expense of added curve smoothing time.

We also showed that a piecewise linear representation of RNA-seq coverage can be used to analyze systematic biases. Our example involved a well-known problem of aligning reads across splice sites. The PPC representation of coverage reveals the magnitude of this bias and suggests that using splice libraries during the alignment procedure need not necessary eliminate it completely.

Other applications of piecewise polynomial curves are possible. In particular, wherever piecewise constant (i.e. run-length encoded) curves are used, it is natural to ask whether benefit in terms of compression or interpretability might be drawn from a representation using first- or higher-order polynomials. Signals encoded by PPCs need not be limited to the types above; they can just as well be SNP data, binding affinities, normalization factors, probability mass functions, or any other function in one dimension.

## Methods

**Figure 7:**
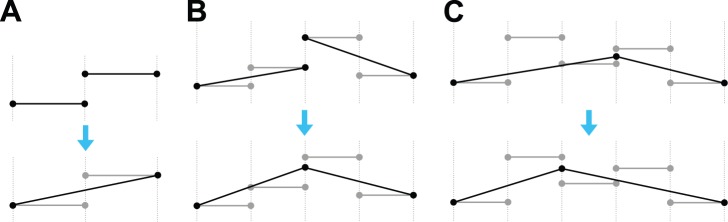
Operations on knots. Gray horizontal segments represent original data, black segments represent the PPC representation. Vertical lines show positions of knots and need not be evenly spaced in actual data. (A) Knot removal. (B) Adjustment that removes a discontinuity. (C) Adjustment of a knot position.

Our algorithm works from the bottom up, i.e. it starts with an input with a large number of segments and then joins pairs of these together. This produces a more compact description of the data in which the fused segments are represented by polynomial functions. In brief (see Text S1 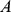 for technical and implementation details), the algorithm starts by reading a run-length encoded input signal from disk. We call the start positions of these runs, borrowing from the literature on splines, as ‘knots.’ The algorithm marks all knots as candidates for processing and visits them in a random order. At each knot, the algorithm attempts to remove the knot by replacing the segments adjacent to it by a single longer segment (Figure 7A), by adjusting the endpoints of the adjacent segments (Figure 7B), or by moving the location of the knot (Figure 7C), in this order. Once one such pass over all the knots is complete, the whole process starts again, but only visiting those knots that have been modified in the previous pass or whose immediate neighbors were. The procedure ends either when all knots are removed or when no further changes to the PPC are consistent with the adjustment criteria. The PPC model is finally output to disk in a tabular format showing the segment lengths and the polynomial coefficients.

Knot operations (Figure 7) involve generating a new polynomial line fitting the original data and in some cases this is aided by a local error minimization calculation. Since we work with curves rather than collections of points, we define local error as an integral of the squared difference between curves (this is a difference between our approach and traditional point-based regression techniques.) Although we sometimes minimize error to produce well-fitting polynomials, we stress this is only performed locally. The approach does not guarantee or even attempt to find a globally optimal fit.

Whether a given knot operation is carried out or rejected depends on the smoothing “method” and an associated numerical parameter 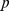. We implemented a number of distinct “methods” to make the tool applicable in a range of applications. These methods compare areas 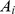 under the original segments, labelled by an index 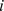, of the input curve and the corresponding areas 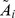 under polynomials.

One method accepts knot changes as long as the relative differences in areas below the original run-length encoded signal and corresponding sections of the PPC are below a threshold, i.e. if the condition 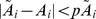 holds for all 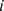. Another method is motivated by the Poisson distribution. It accepts changes to the PPC as long as the areas satisfy the condition 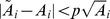 for all 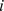, where 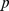 can be used to modulate under- or over-dispersion of the distribution. Both methods are suitable for data originating from high-throughput sequencing. Finally, the last method is based on an empirical t-test statistic computed from segments adjacent to the knot, with 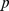 acting as a p-value cutoff (Text S1 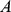). This method is suitable for copy-number detection in array CGH or read-based sequencing of DNA. In all the methods, the focus is on checking the signal conforms to a set of criteria specified by the user and distinguishes the algorithm from canonical spline-building approaches.

A corollary to the tune-ability of the acceptance of knot operations is that the algorithm can be run in a continuum between two limits: a strict limit in which the PPC output is identical to the input, and a loose limit in which the output signal contains only one polynomial segment. Between these limits, the procedure can produce smooth, discontinuous, as well as mixed models.

Because the order of knot adjustment is randomized, repeated application of the algorithm on a given input can produce slightly different results. This has a number of positive effects. First, randomization implies that very little computation is required to determine which knot should be adjusted at any one stage. Second, the variability in the results can be regarded as a measure on uncertainty in the resulting model. Finally, stochasticity enables our approach to sample a fairly large space of possible solutions and is less prone to produce sub-optimal models than a method using steepest descent. We note however that our approach is not a full Monte-Carlo method as it terminates as soon as a locally optimal PPC model is found.

As for implementation, our approach stipulates random access to all knots and efficient removal of any knot. We achieved these requirements using a data structure crossing a hash map and linked list. The hybrid structure requires more book-keeping than a simple array, but pays off in knot-removal performance. The smoothing program completes up to 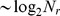 passes, where 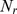 is the number of segments in the original data. In each pass, it compares at most 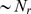 data elements so its asymptotic complexity is 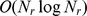. For genomic data, it can be the case that 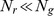 so this is a significant optimization over any method not exploiting run-length encoding.

In practice, the program can create a PPC model for a signal originating from an array (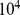 probes) in less that 0.5 seconds on a personal computer with 3.2Ghz processor. Processing a human RNA-seq sample takes about 10 seconds per chromosome. Processing of a human whole-genome sequencing coverage track can take up to 15 minutes per chromosome. For the full-genome signals, the program can require access to 16GB of RAM.

## Supporting Information

Text S1 **Details on algorithm and additional results and examples.**
(PDF)
