# Supplementary material for: Piecewise Polynomial Representations of Genomic Tracks
Source: PLoS One. 2012 Nov 15;7(11):e48941. doi: 10.1371/journal.pone.0048941 (PMC3499510; doi:10.1371/journal.pone.0048941)
Supplement: Text S1 — Details on algorithm and additional results and examples. (PDF) [file pone.0048941.s001.pdf]

Supplementary Material: **Piecewise polynomial representations of genomic tracks**

Maxime Tarabichi[1], Vincent Detours[1,2], and Tomasz Konopka[1]

[1] *IRIBHM, Université Libre de Bruxelles, Brussels, Belgium*
[2] *Welbio, Université Libre de Bruxelles, Brussels, Belgium*

A Supplementary methods

In this section, we describe the smoothing process in more detail. We consider as input a piecewise constant curve. Such a curve is characterized by a set of positions called knots (a starting position, an end position, and intermediate locations where the value of the curve changes discontinuously) and the value (height) of the curve on each piecewise constant section. We will refer to the intervals between knots as sections or segments. We begin by defining a new piecewise polynomial curve (PPC), which we initially set equal to the input data. It therefore has the same number of knots as the original and all its sections are piecewise constant. The rest of the procedure runs in passes. For the first pass, we mark all knots as potential targets for processing.

In each pass, we visit flagged knots in a random order and, on each one, attempt to perform two operations. The preferred operation is to remove the knot and replace its neighboring sections by a single segment. This option is implemented if and only if the resulting section does not excessively deviate from the original curve. If this change occurs, we flag neighboring knots for processing in the next pass.

The alternative operation is to adjust segments neighboring a knot without removing the knot itself. This is useful, for example, when removing discontinuities in the curve. As in the previous case, this option is taken if and only if the result does not differ too much from the original curve. If it is taken, we flag the neighboring knots as well as the knot itself for processing in the the next pass.

If neither operation succeeds, the knot is marked as unchangeable. After a pass completes, the procedure restarts using the currently flagged set of knots and ends when the set is empty. As the number of flagged knots usually decreases in each pass, the process finishes after a relatively small number of iterations.

The above algorithm constitutes a sub-part of the whole program. Indeed, we repeat this process several times. At first, we attempt to construct a new 'curve' that is piecewise flat like the original. In the second and third rounds, we allow the curve segments to be linear and quadratic (the number of these rounds actually performed depends on the maximal polynomial order specified by the user.) We adopted such a layered approach primarily in order to ensure that quadratic fits are not attempted before all linear possibilities are attempted.

The remainder of this section introduces some notation, derives formulae for computing the polynomial fits, and describes the criteria used in these procedures.

A.1 Notation

Consider a piecewise constant curve divided into N sections. Denote the starting positions and values of the sections as x_i and b_i, respectively, with $i = 0, 1, 2, \dots N-1$. Also, let x_N stand for the curve end point. Without loss of generality, we choose a coordinate system with $x_0 = 0$ and thus obtain $x_N = L$, which we call the 'length' of the curve. We also denote the set of starting indeces as $I = \{0, 1, 2, \dots, N-1\}$.

For each pair of neighboring knots $(i, i+1)$, or equivalently for each curve section i, we define

$$\Delta_{in} = x_{i+1}^n - x_i^n. \tag{1}$$

The simple sum over the set I is

$$\sum_{i \in I} \Delta_{in} = L^n. \tag{2}$$

We also consider the weighted sum of Δ_{in}, with weights b_i,

$$\Gamma_n = \sum_{i\in I} b_i \Delta_{in}. \tag{3}$$

In this notation, the area under each section i is $b_i\Delta_{i1}$ and the total area under several sections is Γ_1.

We will be interested in polynomials of order at most equal to two, i.e.

$$f(x) = a_0 + a_1 x + a_2 x^2. \tag{4}$$

The squared error between this polynomial and the piecewise constant curves is defined as

$$\begin{aligned}
\mathcal{E}^2 &= \sum_{i\in I} \int_{x_i}^{x_{i+1}} \left((a_0 - b_i) + a_1 x + a_2 x^2\right)^2 \, \mathrm{d}x \\
&= \left(a_0^2 L - 2a_0\Gamma_1\right) + \left(a_0 a_1 L^2 + \frac{1}{3}a_1^2 L^3 - a_1\Gamma_2\right) \\
&\quad + \left(\frac{2}{3}a_0 a_2 L^3 + \frac{1}{2}a_1 a_2 L^4 + \frac{1}{5}a_2^2 L^5 - \frac{2}{3}a_2\Gamma_3\right) + \sum_{i\in I} b_i^2 \Delta_{i1}.
\end{aligned} \tag{5}$$

For future convenience, the terms are organized to separate the higher order coefficients a_1 and a_2.

A.2 Removing knots

Every non-boundary knot of a piecewise polynomial curve has one polynomial section on its immediate left and another on its immediate right, each of which can be composed of one or more sections of the original curve. We now discuss strategies for merging two neighboring sections together.

Following the notation from section A.1, we define a set I and corresponding x_i for the starting positions of the piecewise constant sections within the new interval. We then define a polynomial

$$f(x) = a_0 + a_1 x + a_2 x^2 \tag{6}$$

and set the coordinate system so the leftmost position of the interval is $x = 0$ and the rightmost position is $x = L$. We require that the polynomial preserve the existing boundary conditions at $x = 0$ and $x = L$. Thus we impose

$$f(0) = a_0 = F \tag{7a}$$

$$f(L) = a_0 + a_1 L + a_2 L^2 = G, \tag{7b}$$

where F and G are constants fixed by the two endpoints of the segments that we want to join together. Enforcing these boundary conditions is an important step in our procedure because it ensures that when a continuous curve with a large number of segments is processed, the outcome is also continuous.

Further details of the polynomial fitting depend on the orders of the previous polynomials on the two sections. At first, we attempt to use a polynomial or order equal to the maximum of the orders on the old sections. If this is not possible (see criteria in Section A.5), we attempt to use a polynomial with an order incremented by one, but not greater than two. If this fails as well, we give up on knot removal. This procedure guarantees that the smoothed curve is less "overfitted" than the original and in particular avoids joining two piecewise constant sections by a quadratic polynomial. Some of the situations that may arise and their smoothings are shown in Figure 1.

To order 1

When the required polynomial order is one (Figures 1b and 1c), we use equation (6) and immediately set $a_2 = 0$. The boundary conditions then become a set of two linear equations in two unknowns with the solution given by

$$a_0 = F \tag{8a}$$

$$a_1 = \frac{G - F}{L}. \tag{8b}$$

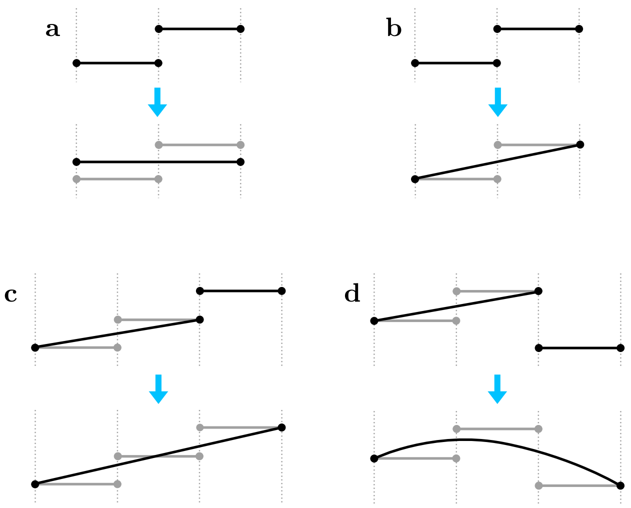

Figure 1: **Knot removal.** (a-d) Sample portions of piecewise polynomial curves with two segments and the strategies used for merging them. Gray segments show the piecewise constant pieces of the original curve. Black segments are segments of the smoothed curve. Vertical dashed lines are isoposition guides.

To order 2

For quadratic fits (Figure 1d), we consider equation (6) but allow all three parameters a_0, a_1, and a_2 to be nonzero. To determine the solution uniquely, we minimize the error (5) with respect to a_2. This involves setting a derivative to zero,

$$\frac{\partial \mathcal{E}^2}{\partial a_2} = \frac{2}{3}a_0 L^3 - \frac{2}{3}\Gamma_3 + \frac{1}{2}a_1 L^4 + \frac{2}{5}a_2 L^5 = 0. \tag{9}$$

Together with the boundary conditions, this leads to the solution

$$a_0 = F \tag{10a}$$

$$a_1 = \frac{G-F}{L} - a_2 L \tag{10b}$$

$$a_2 = \frac{10}{L^5}\left(\frac{1}{6}FL^3 - \frac{2}{3}\Gamma_3 + \frac{G}{2}L^3\right). \tag{10c}$$

Importantly, this operation is not attempted in cases where the two segments being joined are both order 0.

To order 0

The case with order 0 (Figure 1a) is slightly different because it implies setting $a_1 = 0$ and $a_2 = 0$ in (6). We are thus free to adjust only one parameter, a_0, and it becomes impossible to simultaneously enforce continuity both the left and right boundary conditions. We thus abandon both continuity conditions and resort to minimization of squared error. This involves setting

$$\frac{\mathrm{d}\mathcal{E}^2}{\mathrm{d}a_0} = 2a_0 L - 2\Gamma_1 = 0, \tag{11}$$

which gives

$$a_0 = \frac{\Gamma_1}{L}. \tag{12}$$

Note that in this equation, the right hand side is defined in terms of the weighted sum (3) over the, perhaps numerous, piecewise constant sections of the original curve that fall within the interval of interest. However, because of linearity and the fact that this computation is invoked only when the left and right sections of the smoothed curve are themselves constants, it can be shown that Γ_1 can be equivalently computed using the two constant sections from the smoothed representation.

A.3 Adjusting knots

If a knot cannot be removed entirely, we attempt to adjust the polynomials on its left and right in order to remove a discontinuity at the knot and minimize the error between the smoothed and the original curves. Some examples of such smoothings are shown in Figure 2. In all cases, we only consider adjusting coefficients of existing polynomials and never consider increasing the order of a polynomial.

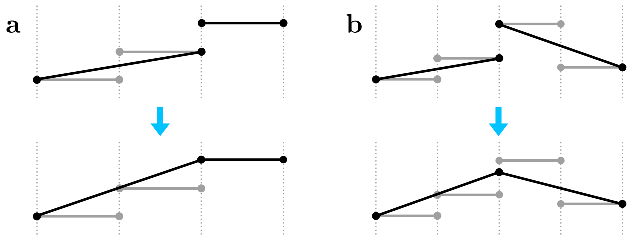

Figure 2: **Discontinuity removal.** (a-b) Portions of piecewise polynomial curves exhibiting discontinuities, and the adjustments that can be made on the neighboring segments to remove them. Gray segments show the piecewise constant pieces of the original curve. Black segments are segments of the smoothed curve.

We describe the left and right neighborhoods using I and I', respectively. Generally, we use unprimed and primed variables to describe quantities on the left and on the right. We denote the polynomial equations for the two segments as[1]

$$f(x) = a_0 + a_1 x + a_2 x^2 \tag{13a}$$
$$f'(x') = a'_0 + a'_1 x' + a'_2 x'^2. \tag{13b}$$

Importantly, we use different coordinate systems on the two regions, and set the origin in each as the left-most position (section A.1) in that section. We impose boundary conditions through the constraints

$$f(0) = a_0 = F, \tag{14}$$
$$f'(L') = G, \tag{15}$$

and ask for continuity at the knot position by requiring

$$f(L) = f'(0). \tag{16}$$

Orders (0,0)

If the existing segments on the left and right are both of order zero and there is a discontinuity at the knot, it is impossible to perform a boundary preserving smoothing without changing the orders of the segments. This situation is thus

Orders (1,0), (2,0), (0,1), and (0,2)

If the right segment is constant and the left segment is linear or quadratic (cases (1,0) and (2,0)), the only way to remove any discontinuity is to adjust the end level of the left polynomial (Figure 2a). The smoothing problem is then reduced to one of the type discussed in section A.2. That is, the left segment is set using (8) or (10) in the linear and quadratic cases, respectively. The right segment is unchanged.

When the left segments is constant (cases (0,1) and (0,2)), the situation is almost equivalent and formulae (8) and (10) are again suitable, except that they are applied on the right segment.

Orders (1,1)

If both left and right segments are linear, they may be adjusted to make the endpoint of the left segment match the start point of the right segment (Figure 2b). In this case we have $a_2 = 0$ and $a'_2 = 0$ and the boundary and continuity conditions are

$$f(0) = a_0 = F, \tag{17a}$$
$$f(L) = f'(0) = a_0 + a_1 L = a'_0, \tag{17b}$$
$$f'(L') = a'_0 + a'_1 L' = G. \tag{17c}$$

[1] The notation $f'(x')$ describes a polynomial on the right interval and does not imply a derivative with respect to x'.

There is one free parameter, so we set out to minimize the total squared error over the two segments,

$$\mathcal{E}_{tot}^2 = \mathcal{E}_I^2 + \mathcal{E}_{I'}^2. \tag{18}$$

Differentiating with respect to a_0', we set

$$\frac{\mathrm{d}\mathcal{E}_{tot}^2}{\mathrm{d}a_0'} = \frac{\partial \mathcal{E}_{tot}^2}{\partial a_0'} + \frac{\partial \mathcal{E}_{tot}^2}{\partial a_1}\frac{\partial a_1}{\partial a_0'} + \frac{\partial \mathcal{E}_{tot}^2}{\partial a_1'}\frac{\partial a_1'}{\partial a_0'} = 0. \tag{19}$$

Using this together with

$$\frac{\partial a_1}{\partial a_0'} = \frac{1}{L}, \quad \text{and} \quad \frac{\partial a_1'}{\partial a_0'} = -\frac{1}{L'}, \tag{20}$$

which follow from (17), we obtain the solution

$$a_0 = F, \tag{21a}$$

$$a_0' = -\frac{3}{2}\frac{1}{L+L'}\left(-2\Gamma_1' + \frac{1}{3}FL' - \frac{\Gamma_2}{L} + \frac{\Gamma_2'}{L'}\right) - \frac{1}{2}F\frac{L}{L+L'}, \tag{21b}$$

$$a_1 = \frac{a_0' - a_0}{L}, \tag{21c}$$

$$a_1' = \frac{G - a_0'}{L'}. \tag{21d}$$

Orders (1,2)

In this case, the boundary and continuity conditions are

$$f(0) = a_0 = F, \tag{22a}$$

$$f(L) = f'(0) = a_0 + a_1 L = a_0', \tag{22b}$$

$$f'(L') = a_0' + a_1' L' + a_2' L'^2 = G, \tag{22c}$$

We treat a_2' and a_0' as independent variables. The other unknowns become dependent variables, so we have

$$\frac{\partial a_1}{\partial a_2'} = 0, \quad \frac{\partial a_1}{\partial a_0'} = \frac{1}{L}, \quad \frac{\partial a_1'}{\partial a_2'} = -L', \quad \text{and} \quad \frac{\partial a_1'}{\partial a_0'} = -\frac{1}{L'}. \tag{23}$$

Minimizing the total error with respect to the independent variables leads us to the following system of linear equations

$$\begin{bmatrix} L & -1 & 0 & 0 \\ 0 & 1 & L' & L'^2 \\ \frac{2}{3}L^2 & L' & \frac{1}{3}L'^2 & \frac{1}{6}L'^3 \\ 0 & -\frac{1}{3}L'^2 & -\frac{1}{6}L'^3 & -\frac{1}{10}L'^4 \end{bmatrix} \begin{bmatrix} a_1 \\ a_0' \\ a_1' \\ a_2' \end{bmatrix} = \begin{bmatrix} -F \\ G \\ -FL + 2\Gamma_1' + \frac{\Gamma_2}{L} - \frac{\Gamma_2'}{L'} \\ \frac{2}{3}\frac{\Gamma_3'}{L'} - \Gamma_2' \end{bmatrix}. \tag{24}$$

As the system of equations is fairly complicated, we do not write the solution explicitly. Rather, in practice we construct matrices with the appropriate values and solve the system numerically.

Orders (2,1)

This is closely related to the previous case. The boundary and continuity conditions are

$$f(0) = a_0 = F, \tag{25a}$$

$$f(L) = f'(0) = a_0 + a_1 L + a_2 L^2 = a_0', \tag{25b}$$

$$f'(L') = a_0' + a_1' L' = G, \tag{25c}$$

We treat a_2 and a_0' as independent variables. We then have

$$\frac{\partial a_1}{\partial a_2} = -L, \quad \frac{\partial a_1}{\partial a_0'} = \frac{1}{L}, \quad \frac{\partial a_1'}{\partial a_2} = 0, \quad \text{and} \quad \frac{\partial a_1'}{\partial a_0'} = -\frac{1}{L'}. \tag{26}$$

Minimizing the total error, we obtain the following system of linear equations in four unknowns

$$\begin{bmatrix} L & L^2 & -1 & 0 \\ 0 & 0 & 1 & L' \\ -\frac{1}{6}L^4 & -\frac{1}{10}L^5 & 0 & 0 \\ \frac{2}{3}L^2 & \frac{1}{2}L^3 & L' & \frac{1}{3}L'^2 \end{bmatrix} \begin{bmatrix} a_1 \\ a_2 \\ a'_0 \\ a'_1 \end{bmatrix} = \begin{bmatrix} -F \\ G \\ \frac{1}{3}FL^3 + \frac{2}{3}\Gamma_3 - \Gamma_2 L \\ -FL + 2\Gamma'_1 + \frac{\Gamma_2}{L} - \frac{\Gamma'_2}{L'} \end{bmatrix}. \tag{27}$$

As in the previous case, we solve for the unknown variables numerically.

Orders (2,2)

The boundary and continuity conditions become

$$f(0) = a_0 = F, \tag{28a}$$
$$f(L) = f'(0) = a_0 + a_1 L + a_2 L^2 = a'_0, \tag{28b}$$
$$f'(L') = a'_0 + a'_1 L' + a'_2 L'^2 = G, \tag{28c}$$

We select a'_0, a_2, and a'_2 to represent the three degrees of freedom. We then find

$$\frac{\partial a_1}{\partial a'_0} = \frac{1}{L}, \qquad \frac{\partial a_1}{\partial a_2} = -L, \qquad \frac{\partial a_1}{\partial a'_2} = 0, \tag{29a}$$

and

$$\frac{\partial a'_1}{\partial a'_0} = -\frac{1}{L'}, \qquad \frac{\partial a'_1}{\partial a_2} = 0, \qquad \frac{\partial a'_1}{\partial a'_2} = -L'. \tag{29b}$$

Minimizing the total error with respect to the three independent variables, we obtain the following system of equations

$$\begin{bmatrix} L & L^2 & -1 & 0 & 0 \\ 0 & 0 & 1 & L' & L'^2 \\ \frac{2}{3}L^2 & \frac{1}{2}L^3 & L' & \frac{1}{3}L'^2 & \frac{1}{6}L'^3 \\ -\frac{1}{6}L^4 & -\frac{1}{10}L^5 & 0 & 0 & 0 \\ 0 & 0 & -\frac{1}{3}L'^3 & -\frac{1}{6}L'^4 & -\frac{1}{10}L'^5 \end{bmatrix} \begin{bmatrix} a_1 \\ a_2 \\ a'_0 \\ a'_1 \\ a'_2 \end{bmatrix} = \begin{bmatrix} -F \\ G \\ -FL + 2\Gamma'_1 + \frac{\Gamma_2}{L} - \frac{\Gamma'_2}{L'} \\ \frac{1}{3}FL^3 + \frac{2}{3}\Gamma_3 - \Gamma_2 L \\ \frac{2}{3}\Gamma'_3 - \Gamma'_2 L' \end{bmatrix}. \tag{30}$$

As in the previous cases, we solve this system numerically.

A.4 Moving knots

The knot removal and adjustment strategies above do not guarantee that the formed PPC is an optimal description of the original data. In order to fine-tune the smoothed PPC, we implement an optional knot-moving stage. When enabled, we consider adjusting not only the vertical position of a knot but also its location along the curve (Figure 3).

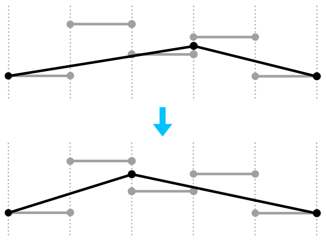

Figure 3: **Knot moving.** Gray segments show the piecewise constant pieces of the original curve. Black segments are segments of the smoothed curve. Knot moving changes the position of one of the knots only while preserving the location of the segment endpoints and the segment orders.

We compute the error between the original data and the two segments neighboring the knot. We repeat the calculation after moving the knot location to the left by one segment of the original PPC, then by two, and so on up to a user-specified maximum. We also repeat the calculation moving to the knot toward the right. Finally, we set the knot at the position that minimizes the error.

A.5 Acceptance criteria

The above strategies provide suggestions for replacing one description of the piecewise polynomial curve by another one. Before implementing the removal or smoothing actions, however, the suggestions need to be checked to ensure that the resulting curve does not deviate too much from the input data. To this extent, we consider the relevant interval I of the original piecewise constant curve and compute the areas $A_i = b_i \Delta_{i1}$ (see section A.1). We also compute the areas $\tilde{A}_i$ under the suggested polynomial segment over the matching regions. We then use these areas to reach a decision about the suggested operation using one of several methods. The method and an associated numerical parameter p are user-specified.

A.5.1 Poisson

In the Poisson acceptance scheme, we compare the paired original and proposed areas pairwise. We accept the suggested segment if all pairs satisfy

$$|\tilde{A}_i - A_i| < p\sqrt{\tilde{A}_i}. \tag{31}$$

The parameter p (defaults to 1) is useful for changing the aggressiveness of the smoothing. The default value of 1 is suitable when A_i are Poisson random variables and higher values can be used to achieve greater compression. Values lower than 1 can be useful when the processed signal encodes Poisson random variables which have been normalized (e.g. to represent a probability scale).

It is worth stressing that the above test condition is applied on areas under segments. The consequence of this can be understood via an example. Consider two sections of an input signal with length 1 and 2 respectively, both with value 30. Such sections can be frequently encountered in sequencing coverage data. Consider a piecewise-flat segment covering these two sections having value 26. The areas compared for the shorter segment will be $30 (= 30 \cdot 1)$ and $26 (= 26 \cdot 1)$, which will pass the acceptance test for $p = 1$ ($|26 - 30| < \sqrt{26}$). For the longer section, the areas compared will be $60 (= 30 \cdot 2)$ and $52 (= 26 \cdot 2)$, which result in a failed acceptance test ($|52 - 60| > \sqrt{52}$). In a signal with heterogeneous section lengths, this means that knot removal and smoothing operations occur less frequently around long sections.

A.5.2 Percent

For the percentage-based acceptance scheme, we check that all area pairs satisfy

$$|\tilde{A}_i - A_i| < p\tilde{A}_i. \tag{32}$$

In this method, the parameter p (defaults to 0.1) is a threshold for the relative difference in areas. Compared to the Poisson method, this criterion gives equal treatment to sections of all lengths.

A.5.3 t-test

The t-test acceptance scheme is designed for use with piecewise-constant smoothings and works quite differently from the previous schemes. When a new segment is proposed, for example by joining two PPC segments together, we do not verify that the original data is consistent with the new segment. Instead, we compute a t-test like score which determines whether or not the two PPC segments are significantly different to warrant keeping them separated. In practice, we first assign weights to the original areas according to the lengths of the original segments. Then, we compute the means μ, μ' and standard deviations σ, σ' of the data on the left and right PPC segments. The t-test like score is then defined as

$$z = \frac{\mu - \mu'}{\widetilde{\sigma}}, \quad \text{where} \quad \widetilde{\sigma} = \left[\frac{\sigma^2}{(L-1)^2} + \frac{\sigma'^2}{(L'-1)^2} \right]^{1/2}. \tag{33}$$

To determine whether a value of z is significant, we permute the areas $N = (L + L')^2$ times (at most $10/p$ times) and repeat the above calculation to obtain a reference distribution of scores. This distribution is used to assign a p-value to z. A knot removal operation is accepted if the p-value is above a threshold set by the parameter p (defaults to 0.01), and rejected otherwise.

This test becomes active when both left and right segments contain at least two sections of the original data. If one of the sections contains fewer than two elements, a knot removal operation is automatically accepted.

A.6 Other Issues

Subdivision into windows

Very long curves can take a large amount of memory and take a long time to process. To improve efficiency, we note that curve sections with values of zero are immutable as changes to such segments are rejected by our acceptance criteria (Section A.5. This is not true for the t-test.). Thus, we divide curves into windows separated by long zero-level pieces and then process each of these windows independently (the length of the zero-level run can be set by the user). The windows are patched together at the end.

Boundary knots

In practice, piecewise polynomial curves are defined over compact intervals and this implies that two knots define the curve boundaries. (Implicit boundaries also appear when a long curve is split into windows as described above.) In contrast to non-boundary knots, they have only one neighboring curve segment instead of two. Although these knots cannot be removed, they can be smoothed. The procedures defined in the previous section, however, need to be modified to account for the boundaries.

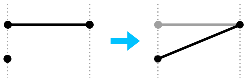

Figure 4: **Discontinuity removal at boundaries.** Boundary knots can have discontinuities just like interior knots.

Dirichlet boundary conditions specify fixed curve values at and beyond the boundary positions. Because we deal with curves with discontinuities, we allow for the possibility of a discontinuity at the boundary knots as well. For example, while the starting position of a first segment might be a certain value, we can in principle fix the left boundary condition to a different value. This trick is handy when splitting a long signal into windows and smoothing the windows independently.

Smoothing accuracy

A knot adjustment suggestion (Section A.3) changes the start and end values of two neighboring segments (Figure 2). When a knot is adjusted, its neighbors are then flagged for adjusting as well. If these knots are smoothed, then the knot we started with is flagged again, and so on. The adjustments normally become very small after a few iterations. We thus monitor the value of the coefficient a'_0 (see Section A.3) before and after the knot adjustment, and reject if the change is smaller than a threshold value. This threshold value is absolute (as in non-relative, not un-changeable) and is set by default to 0.01.

A.7 Comparison to other methods

We compare the outputs generated by our PPC generating algorithm to other smoothing methods in Table 1. One important method missing in the comparison is adaptive spline modeling, i.e. smoothing splines with dynamically determined knots. This actually the method most closely related to our approach, except using Bayesian fitting criteria, maximally smooth polynomials, and a monte-carlo engine for optimizing knot placement. Unfortunately, the implementation we found [6] did not compile and run in our locale. We would expect it to give similar results to our approach, but its more sophisticated criteria would make it less suitable for processing very long signals with many domain changes.

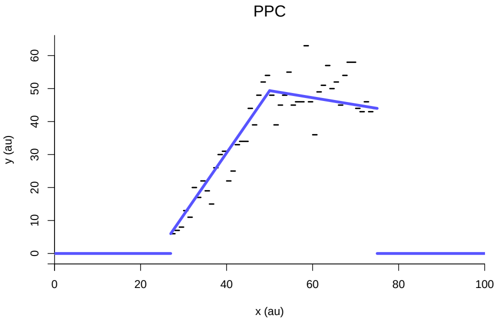

Piecewise polynomials, obtained by our approach. In this case, we used a Poisson error model with parameter $p = 3$. The result is roughly correct, but one segment here shows a slight downward slope whereas it should actually be flat. The result shows a discontinuity at positions 75 and 25. The significance of one but not the other can be determined in post-processing.

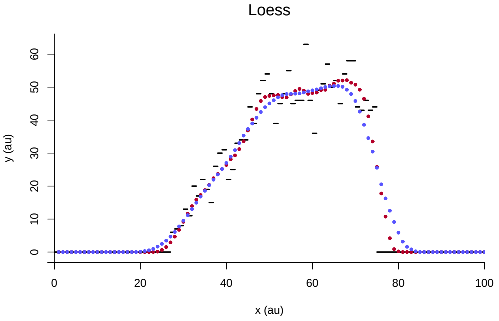

Loess is produced with the `loess` function in `R`. The function requires specification of a parameter determining a local region; the red and blue outputs show different settings. The result is a collection of points, not a functional model. Good smoothing is achieved in already smooth areas, but sharp discontinuities are blurred.

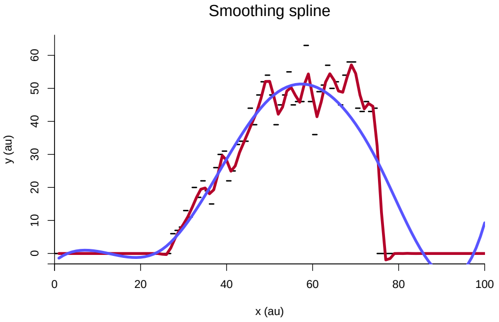

Smoothing splines are produced with the `smooth.spline` function in `R`, which requires specification of the location or the number of knots. The red curve is a result of using all data points as knots. The blue curve uses only 5 knots. The smoothness and continuity conditions induce wiggles near sharp boundaries. These can only be avoided by using a high knot density at such loci.

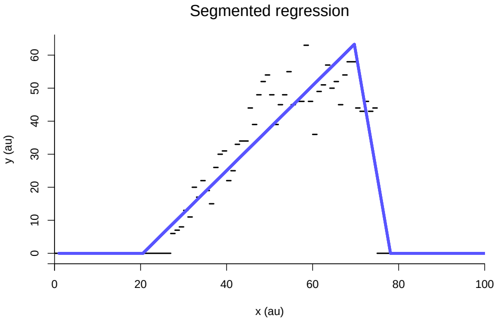

Segmented regression is produced with the `segmented` package in `R`. It is fairly successful, but includes an unnecessary very steep segment on the right. A disadvantage of the algorithm is that it requires defining an initial set of knots and sometimes aborts with no output if this set is not close to optimal.

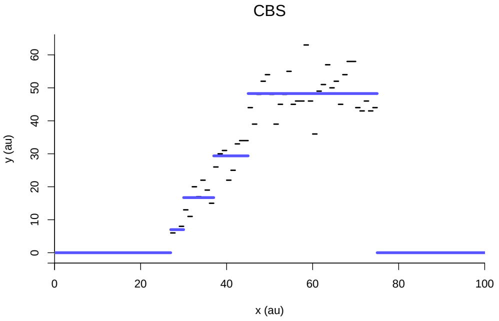

CBS segmentation is produced with the `DNAcopy` package in `R`. The method only creates flat segments. It works very well to detect the plateau, but produces many short stair-like segments on the sloping portion of the signal.

Table 1: **Comparison of methods.** The input signal is trapezium shaped with one gentle and one sharp side. The noise generated is Poisson. Arbitrary units.

B Supplementary tables and figures

In all cases, linear and quadratic smoothing is performed with the `-c` option off (Section C). This means that two piecewise constant segments are never smoothed into constant sections.

B.1 Compression performance

B.1.1 Synthetic Data

			Memory (ICR)		Disk (ICR)		Mean Len.	
**Method**	**P**	**O**	**8r/nt**	**250r/nt**	**8r/nt**	**250r/nt**	**8r/nt**	**250r/nt**
poisson	1	0	3.6	22.1	1.8	11.5	21.3	39.0
poisson	1	1	2.2	13.1	0.9	5.6	22.1	38.2
poisson	1	2	2.1	13.3	1.0	6.5	29.3	54.3
poisson	2	0	8.4	67.8	3.4	32.6	51.0	125.9
poisson	2	1	4.9	30.2	1.8	12.4	48.8	90.1
poisson	2	2	4.8	32.3	2.1	14.6	67.0	135.7
poisson	4	0	23.8	403.0	9.0	236.9	153.9	1256.5
poisson	4	1	10.4	182.1	3.7	78.9	107.0	684.3
poisson	4	2	10.9	199.1	4.3	99.6	157.4	1116.2
percent	0.05	0	0.7	18.5	1.0	9.7	4.3	32.4
percent	0.05	1	0.7	11.1	0.7	4.8	6.5	32.2
percent	0.05	2	0.5	11.1	0.7	5.5	7.4	45.4
percent	0.1	0	1.3	67.3	1.1	32.4	7.6	124.9
percent	0.1	1	1.1	29.6	0.6	12.2	10.8	88.2
percent	0.1	2	1.0	31.7	0.7	14.3	13.8	133.1
percent	0.2	0	3.2	509.6	1.7	361.4	19.2	2021.2
percent	0.2	1	2.3	346.9	1.0	188.2	23.2	1808.8
percent	0.2	2	2.2	326.6	1.1	204.7	30.5	2443.4

Table 2: **Compression due to PPCs - synthetic signals with 50nt reads** Extension of Table 1 from the main text. Columns P and O denote the error model parameter p and the order of the PPCs, respectively. Memory usage to store a signal is computed using the `object.size` function in R. Disk usage is computed using gzip-ed text files. ICR stands for inverse compression ratio. Values in the Mean Len. columns indicate the lengths of a typical segment in the PPCs. For reference, mean lengths in uncompressed signals are 2.8 and 1.1 for the low and high coverage density signals. r/nt stands for reads per nucleotide.

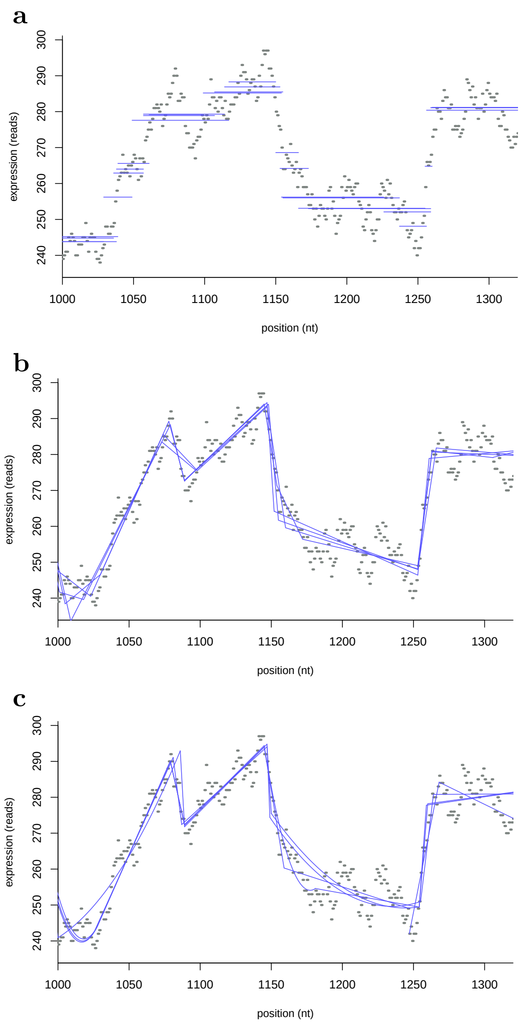

Figure 5: **Multiple smoothing runs on a single high-coverage signal** As the PPC smoothing algorithm is stochastic, it produces slightly different results on each invocation. (a) Piecewise constant smoothing. (b) Linear smoothing. (c) Quadratic smoothing.

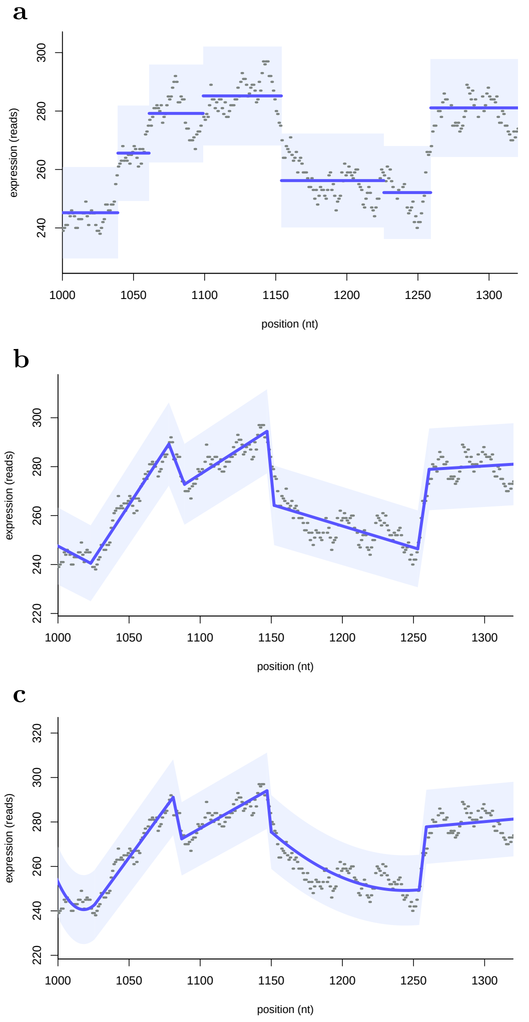

Figure 6: **Smoothing example.** Smoothings using piecewise constant (a), linear (b), and quadratic (c) polynomials.

B.2 Copy-number detection

B.2.1 Array data

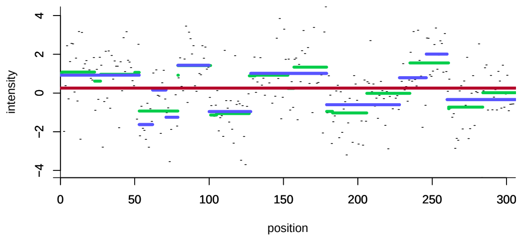

Figure 7: **Low-resolution CGH signals with aberrations.** Dots are probe intensities, horizontal lines indicate true copy-number levels (green), and segments output by CBS (red) and the median of 5 PPC runs (blue). This is a situation in which, due to large overall chromosome variance, CBS is unable to detect distinct segments while the bottom-up PPC approach can.

		Results				
**Probes**	**Approach**	**TP**	**FP**	**TN**	**FN**	**BTP**
300	PPC (1pass)	0.31	0.04	0.52	0.13	0.36
300	PPC (5pass)	0.33	0.03	0.53	0.11	0.38
300	CBS	0.31	0.04	0.52	0.13	0.35
1500	PPC (1pass)	0.40	0.02	0.55	0.02	0.41
1500	PPC (5pass)	0.41	0.02	0.55	0.02	0.42
1500	CBS	0.42	0.02	0.56	0.01	0.42
50000	PPC (1pass)	0.42	0.00	0.57	0.00	0.42
50000	PPC (5pass)	0.42	0.00	0.57	0.00	0.42
50000	CBS	0.42	0.00	0.57	0.00	0.42

Table 3: **Classifier performance on array data.** Array data for the synthetic array signals were segmented using PPCs and CBS. For the PPC method, we prepared a segmentation from a single smoothing run (1 pass) as well as a median of five passes (5 pass). We assigned each PPC and CBS segment a label of CN abnormality if more than half of the width overlapped with a true CN event (in this calculation, signals with multiple events were pooled together). The dataset was divided into a part for training (85%) and testing (15%) and SVMs were trained as described in section B.2.2. The SVM predictions on the test set were evaluated by computing true positives (TP), false positive (FP), true negatives (TN), false negatives (FN), and best true positive (BTP, see main text). The measures in the table are ratios of these measures relative to the number of probes in the test set, averaged over 40-fold cross validation. Ratios are comparable within the groups for 300, 1500, and 50000 probe chromosomes, but not across these sets. Results show that PPC segmentation is competitive against the gold standard, especially for the short chromosomes.

Passes	Dataset	Ref.	Method CBS	GLAD	HMM
1	20chromosomes	[7]	0.93	0.66	**0.19**
1	below5	[7]	0.98	0.88	**0.02**
1	5to10	[7]	0.98	0.69	**0.29**
1	10to20	[7]	0.90	0.52	**0.22**
1	above20	[7]	0.91	0.80	**0.15**
1	9 events (300 probes)		**0.38**	**0.08**	**0.08**
1	9 events (1500 probes)		0.76	**0.33**	**0.00**
1	9 events (50,000 probes)		1	0.66	**0**
5	20chromosomes	[7]	0.82	0.53	**0.12**
5	below5	[7]	0.98	0.87	**0.01**
5	5to10	[7]	0.95	0.55	**0.16**
5	10to20	[7]	0.66	**0.31**	**0.09**
5	above20	[7]	0.77	0.67	**0.06**
5	9 events (300probes)		**0.26**	**0.05**	**0.05**
5	9 events (1500probes)		**0.44**	**0.19**	**0.00**
5	9 events (50,000 probes)		1	0.65	**0**

Table 4: **Comparison of PPC with three array segmentation methods.**The table has similar structure to Table 2 in the main text. We considered five previously published datasets [7] and three sets of signals of the type described in the main text. For each dataset, we performed segmentation using PPC and three other methods: CBS [5], GLAD [2] and HMM [1]. Values in the tables are proportions of times the output from a method had lower mean squared error than a segmentation produced via PPC. Instances where PPC was better more often (proportions < 0.5) are highlighted in bold. The results show that CBS and GLAD often give better segmentation than PPC, but that PPC is nonetheless better in a non-negligible fraction of cases. Results for datasets labeled 9 events (described in the main text, an example is in Figure 8) suggests there exist classes of signals not represented in the other datasets for which PPC gives considerably better results than any of the other methods. We thus conclude that PPC is complementary to existing methods.As our algorithm is stochastic, all proportions are approximate with estimated error of about 0.05 (differences in values here and in Table 2 of main text are helpful in understanding variation between runs.) As is common with stochastic algorithms, performance improves when averaging results over multiple runs/passes.

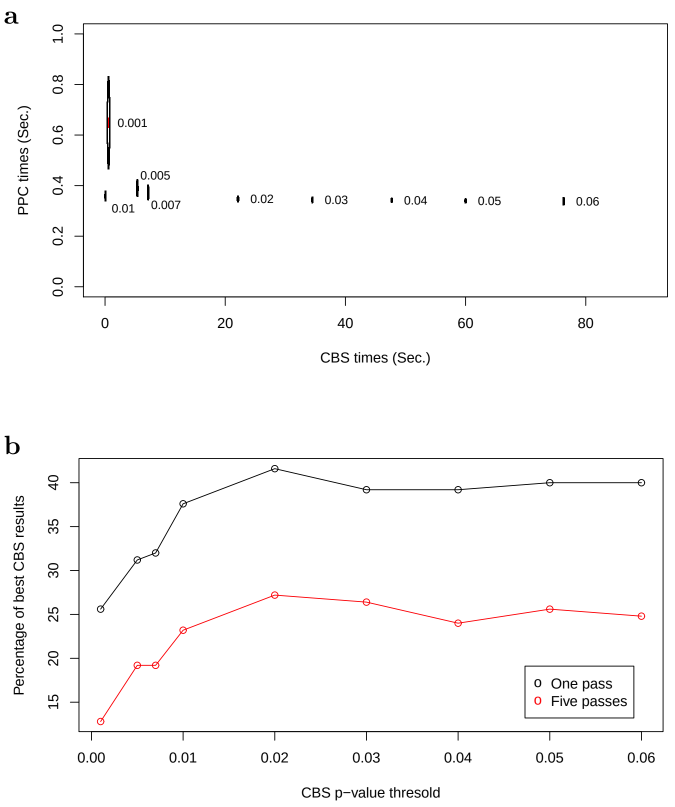

Figure 8: **Effect of p-value settings on segmentation.** The plots show further metrics comparing PPC and CBS segmentation. Both figures are computed on signals of the type described in section 3.2.1 (300 probes and 9 copy-number events) (a) CBS running time increases very quickly with p-value. However, CBS is well-optimized for $p = 0.01$ and completes in comparable time to PPC. Intervals indicate scatter over distinct inputs. (b) This figure is related to Table 2 in the main text. For the short signals with many CN events, the proportion of times CBS segmentation gives lower error compared to PPC is lower than 50%. This observation is robust to changing the p-value used in CBS.

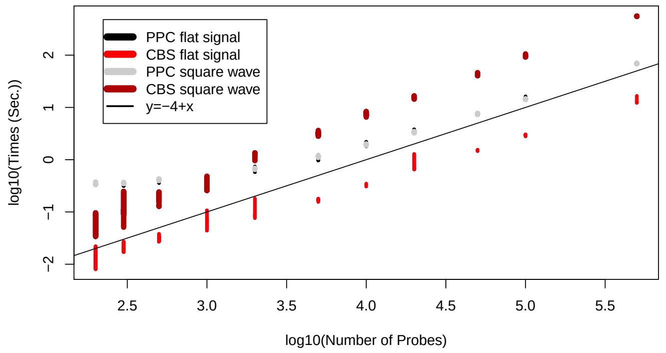

Figure 9: **Running time scaling.** The running times of CBS and PPC approaches scale with the length (number of probes) of the signal. In the bottom-up approach, the running times are roughly equivalent for signals that are flat (with noise) or consist of a square wave (with noise). For the same signals, the top-down approach exhibits a shift in the running times while simultaneously missing many more events in the square wave pattern. All measurements performed with default settings. The diagonal line with unit slope shows linear scaling.

B.2.2 Sequencing data

We tested a number of PPC segmentation strategies by varying the aggressiveness of smoothing via parameter p (Poisson method, p set to 4.5, 5, and 5.5, other values not shown). For comparison, we computed mean coverage in fixed windows (50, 75, and 100bp, other values not shown), converted these means to log-ratios, and treated them using circulary binary segmentation (CBS) [5]. Since neither the PPC curve smoothing or CBS segmentation explicitly call copy number changes, we created classifiers using support vector machines (SVM). The sample was divided into a part for training (8500 chromosomes) and a part for testing (1500 chromosomes). We classified segments output by each approach as copy-number abnormal if more than half of their widths overlapped with the true abnormal region. For the PPC based segmentations, we trained the SVMs using four features: segment width, segment mean, and the means of segments immediately up- and down-stream. The need for features other than segment mean can be understood from Figure 10, which shows that PPC segmentation sometimes produces very short segments above and below the main trend. The classifier must be trained with such information to ignore these local fluctuations. For the CBS based segmentations, we used only two features, segment width and segment mean, as we found this produced better performance.

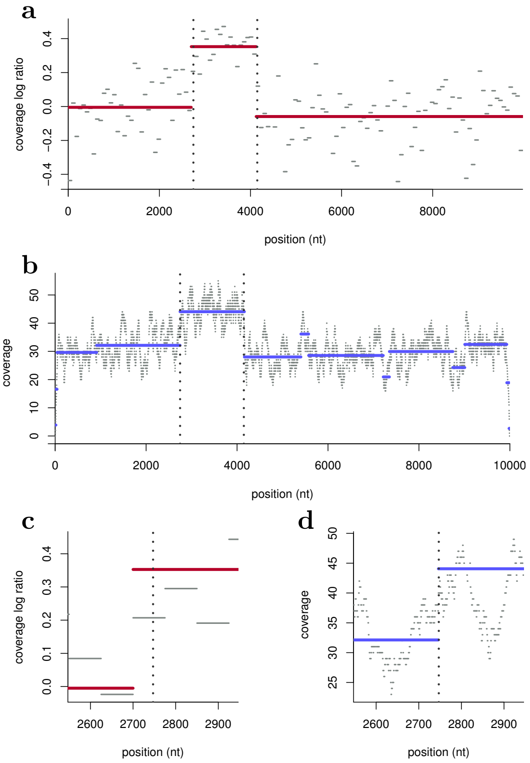

Figure 10: **Signals with copy-number aberration.** Full chromosome views (a,b) and detailed views (c,d) of a signal with a copy-number amplification (between vertical dotted lines). Segmentations were produced by CBS (a,c) with 75bp smoothing windows and our smoothing program (b,d) using Poisson $p = 5$ with knot moving enabled up to 10 segments.

The classification process was repeated 40 times in cross validation (changing sets of training and test chromosomes) by 4 times recomputing smoothing of all curves to investigate the effect of stochasticity in our algorithm. To summarize the performance of each classifier, we computed the number of bases called as copy-number normal and abnormal and compared to the true counts (Table 5). All approaches exhibit high sensitivity ($TP/(TP + FN) > 90\%$) and specificity ($TN/(TN + FP) > 99\%$). The CBS strategy produces best results when the width of the fixed windows are equal to the read length, i.e. autocorrelation length of the signal (75bp). The PPC strategies achieve comparable performance and sometimes show higher true positive rates together with lower false negative rates. We stress this does not arise because the control method misses regions of copy number changes entirely. The reference method is in fact quite reliable, but is limited in detecting breakpoints due to the fixed window length (the example in Figure 10 is picked to demonstrate this point).

Another measure of comparison is the number of best true positives (BTP). This is the maximal

Approach		Results (kbp)				
	W (bp)	TP	FP	TN	FN	BTP
PPC	236	2043	39	12861	56	2055
PPC	289	2041	42	12859	59	2050
PPC	349	2037	48	12852	62	2045
CBS	100	2012	49	12851	88	2020
CBS	75	2039	39	12862	61	2050
CBS	50	2038	42	12858	62	2044

Table 5: **Copy-number detection performance.** Columns denote window median length (W), true positives (TP), false positives (FP), true negatives (TN), false negatives (FN), and best true positive (BTP) for copy-number aberrations. For PPCs, column W shows the median segment length. For CBS, column W shows the length of the windows used before segmentation.

number of positions that could be called as copy-number abnormal given a perfect classifier; it thus provides information about detection ability that is independent of the specific SVM classifier used in these experiments. The measure is at a maximum for the CBS approach when using 75bp windows. Working with dynamically defined PPC segments can give higher BTP rates, and can be improved beyond the results shown at the expense of using shorter but more numerous segments, which can take more time to classify.

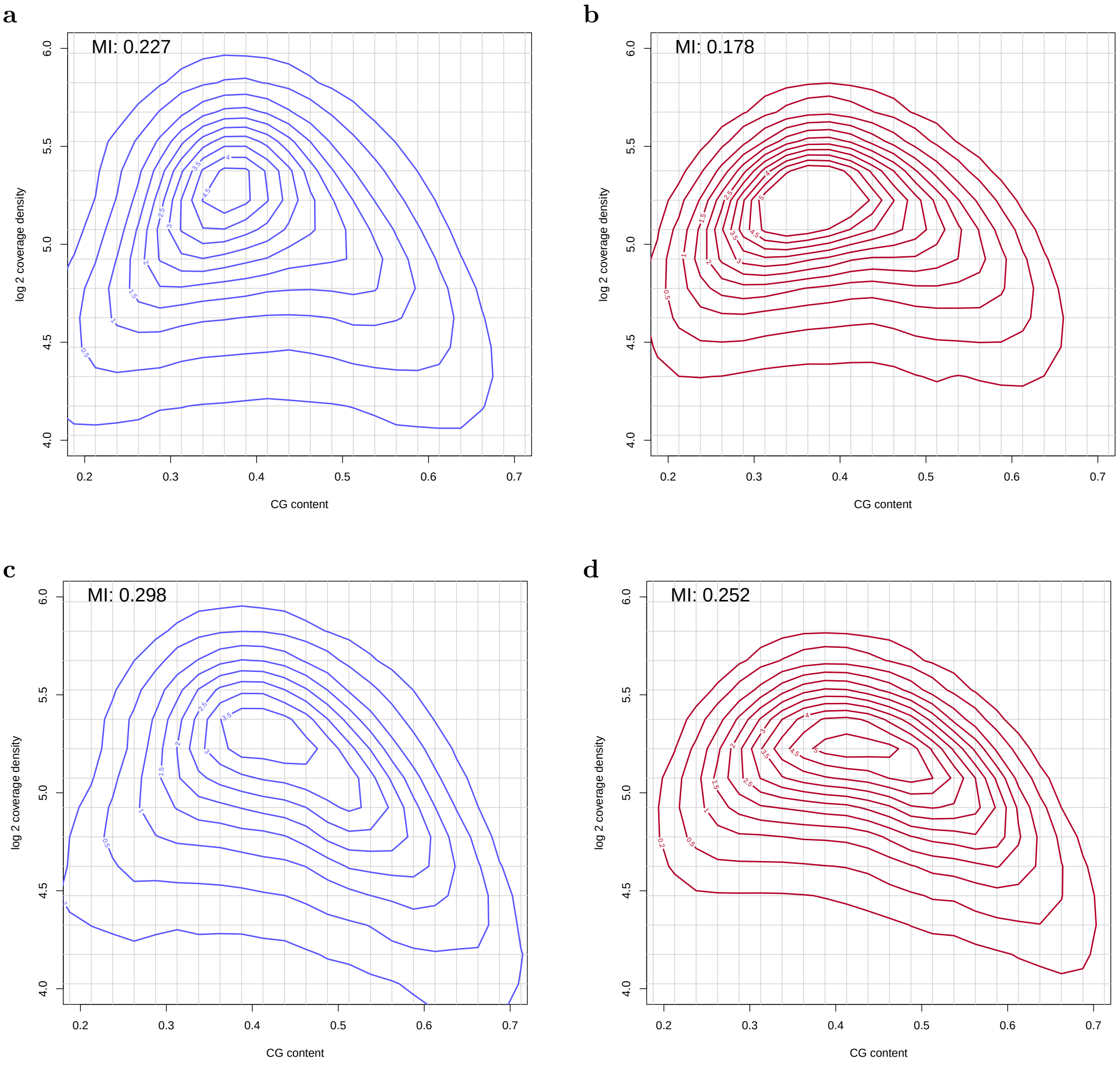

Figure 11: **Mutual information between CG content and coverage density.** Contour plots of distribution of segments on the CG content vs. coverage density plane in sample NA12891 from the pilot phase of the 1000 Genomes Project [4]. (Left) Segments obtained using PPCs. (Right) Segments obtained using a fixed window length. (Top row) Data from chromosome 5. (Bottom row) Data from chromosome 20. Fixed window lengths (98 and 96 for chromosomes 5 and 20, respectively) were picked to match the median segment produced by the PPC procedure. The mutual information (numerical values in top-left corners, computed using the binning shown by the gray grid) is higher when using dynamical segmenting compared to fixed windows of comparable length. Similar differences in MI are present in other chromosomes.

B.3 RNA-Seq

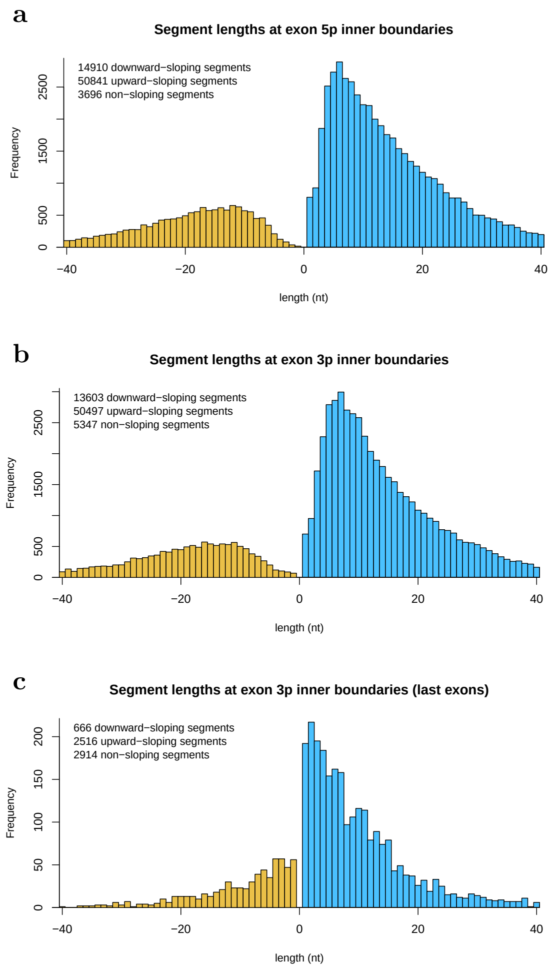

Figure 12: **Segments close to exon boundaries** Extension to Figure 6 in main text. Positive (negative) lengths indicate upward (downward) sloping segments. Colors emphasize sign of slope.

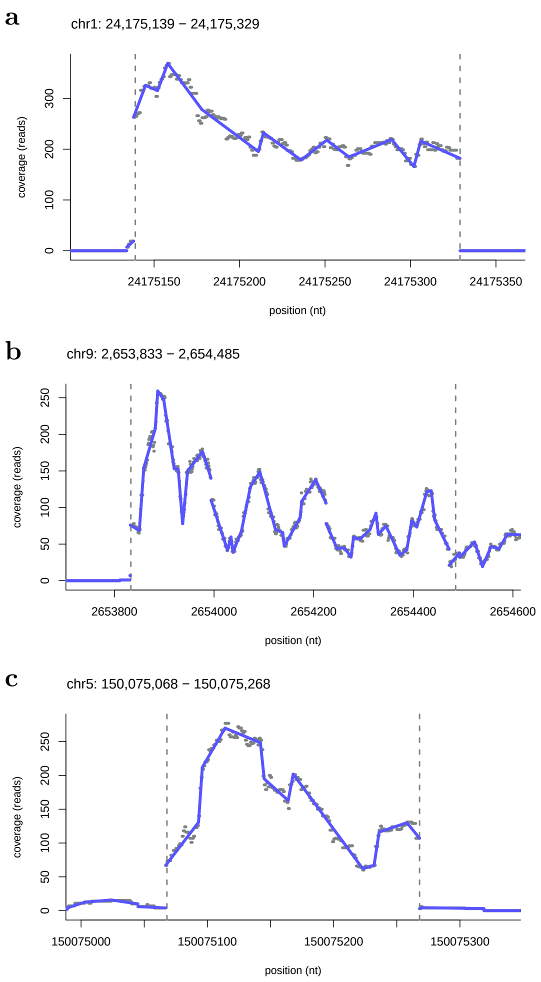

Figure 13: **Smoothing of coverage tracks.** Randomly selected exons with mean coverage density above 50, exon width greater than 100, and where segment slopes near boundaries are (a) positive in inward direction and shorter than 25nt; (b) negative in inward direction and shorter than 25nt; (c) positive in outward direction. Dashed lines indicate exon boundaries. The plots illustrate exon-boundary segments contributing to the dominant parts of the distributions in Fig 12.

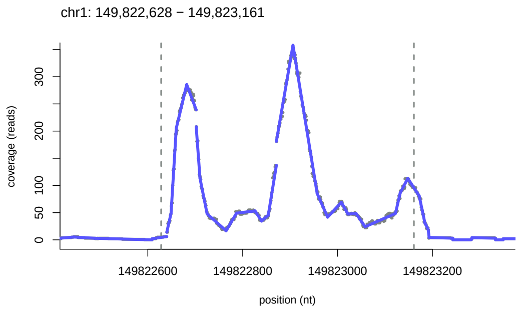

Figure 14: **Smoothing of coverage tracks.** Randomly selected exon with mean coverage density above 50, width greater than 100, and coverage that does not contain a discontinuity within 10 positions away from either boundaries. Dashed lines indicate exon boundaries.

C Software

C.1 Command line parameters

The smoothing program, `locsmoc`, takes some required and some optional parameters/flags (Table 6).

Option	Default	Description
`-f`		[**required**] input/output files. The first instance of `-f` is interpreted as the input file. Input files can be in gzip format (gz extension) or text format (any other extension). The second instance is used as the output destination. Inputs/outputs can be directories, in which case all files contained are processed sequentially.
`-o`	1	order of the smoothed curve. Other accepted values: 0 (use `-c` flag), 2.
`-z`	64	zeros. A value of zero forces all the curve to be smoothed at once. Positive values allow the curve to be split into windows separated by runs with zero value.
`--move`	0	when non-zero, indicates maximal extent of knot moving during (Section A.4)
`--method`	`poisson`	smoothing thresholding method (Section A.5). Other accepted values: `percent, ttest`
`-p`	`1`	smoothing thresholding parameter (Section A.5)
`-a`	`0.01`	accuracy tolerance (Section A.6)
`--seed`	0	seed for random number generation. When set to 0, the random number generator is initialized using the current date as a seed. Otherwise, the specified value is used as the seed.
`-g`	NA	force compress output using gzip
`-c`	NA	consider smoothing into piecewise constant pieces (required when -o=0)
`-h`	NA	input/output files have header rows
`-t`	1	number of threads/processors used

Table 6: **Command line parameters.** Option codes, default values, and descriptions of the command line options to the curve smoothing program. Default value NA indicate the option is a flag that does not requiring a value.

C.2 Examples

The options and flags provide a lot of flexibility. Some of the possibilities are reviewed below.

Input/Output options

To smooth one file using all the default settings, use
`java -jar locsmoc.jar -f infile.txt -f=outfile.txt`

If the input file contains a header row, use
`java -jar locsmoc.jar -h -f infile.txt -f=outfile.txt`

The software can manipulate `gzip` files, so the following generates a compressed outputfile
`java -jar locsmoc.jar -f infile.txt -f outfile.gz`

It is also possible to smooth multiple files at once. This can be done by passing more that two input/output file names

```
java -jar locsmoc.jar -f input1.txt -f output1.txt -f input2.txt -f output2.txt
```

Alternatively, it is possible to specify input/output directories

```
java -jar locsmoc.jar -f inputdir -f outputdir
```

This produces output files with the same names as the input files. The output is compressed whenever the input is. To force compress the output, set the `-g` flag

```
java -jar locsmoc.jar -g -f inputdir -f outputdir
```

Smoothing options

To change the default settings, use the parameters/flags from Table 6.

To change the order of the output piecewise polynomial:

```
java -jar locsmoc.jar -o=[0,1, or 2] -p=0.2 -f infile.txt -f outfile.txt
```

The smoothing method is determined by `--method` and its associated numerical parameter `-p`,

```
java -jar locsmoc.jar --method=percent -p=0.2 -f infile.txt -f outfile.txt
```

For reproducible results, set the random number generator seed (replace the text in [ ] by a numerical value)

```
java -jar locsmoc.jar --seed=[some large integer] -f infile.txt -f outfile.txt
```

Knot-moving is disabled by default, but can be enabled using the `--move` option

```
java -jar locsmoc.jar --method poisson --move 10 -f infile.txt -f outfile.txt
```

C.3 Incorporation in an analysis pipeline

In this section we give a minimalist example of how to use the software to build PPC models for sequencing data. We begin with an alignment file and aim to compute PPCs for the coverage tracks. The example assumes we are working in a UNIX-like environment and have `R` installed with the package `Rsamtools` [3].

We want to compute the coverage track for an alignment in a file `aln.bam`. We can do this in R, so we issue the following commands within the R environment:

```
> library("Rsamtools")
> aln = readGappedAlignment("aln.bam")
> alncov = coverage(aln)
> alncov.chr1 = alncov$chr1
```

The first command loads the `samtools` library for `R`. The second loads the small alignment file we created earlier. The third computes the coverage track and the fourth extracts only the coverage track for chromosome 1. (We assume the chromosomes in the alignment are labelled "chr1," "chr2," etc. If they are not, the last command should be modified accordingly.) The object `alncov.chr1` now holds a run-length encoded signal. We can check this by viewing the object using

```
> alncov.chr1
```

We want to put this signal in a tabular format and save it to disk. We do this as follows

```
> alncov.chr1.table = cbind(runLength(alncov.chr1), runValue(alncov.chr1))
> write.table(alncov.chr1.table, file="aln.chr1.txt", quote=F, row.names=F, col.names=F, sep="\t")
```

We want to compress the text file to save disk space. Then, we want to create two PPC models for the coverage track: one in terms of piecewise flat and the other in terms of piecewise linear segments. We issue the following commands

```
> system("gzip aln.chr1.txt")
> system("java -jar [path-to-locsmoc.jar] --method poisson -p 2 -o 0 -c --move 10
                                  -f aln.chr1.txt.gz -f aln.chr1.o0p2m10.txt.gz")
> system("java -jar [path-to-locsmoc.jar] --method poisson -p 2 -o 1    --move 10
                                  -f aln.chr1.txt.gz -f aln.chr1.o1p2m10.txt.gz")
```

These are actually commands that can be executed on the command line, but we wrap them with `system()` here to perform the full analysis without switching back-and-forth between the command line and `R`. The outputs of the last two commands are files ending with `p2m10.txt.gz`. We can load them into R as normal tables

```
> alncov.chr1.o0p2m10 = read.delim("aln.chr1.o0p2m10", sep="\t", header=F)
> alncov.chr1.o1p2m10 = read.delim("aln.chr1.o1p2m10", sep="\t", header=F)
```

The first of these objects will be a table with two columns: the segment length and the segment level (a_0). The second object will be a table with three columns: the segment length, level (a_0), and slope (a_1).

Information about the `R` session used in this example:

```
> sessionInfo()
R version 2.13.1 (2011-07-08)
Platform: x86_64-pc-linux-gnu (64-bit)

locale:
 [1] LC_CTYPE=en_US.UTF-8       LC_NUMERIC=C
 [3] LC_TIME=en_US.UTF-8        LC_COLLATE=en_US.UTF-8
 [5] LC_MONETARY=C              LC_MESSAGES=en_US.UTF-8
 [7] LC_PAPER=en_US.UTF-8       LC_NAME=C
 [9] LC_ADDRESS=C               LC_TELEPHONE=C
[11] LC_MEASUREMENT=en_US.UTF-8 LC_IDENTIFICATION=C

attached base packages:
[1] stats     graphics  utils     datasets  grDevices methods   base

other attached packages:
[1] Rsamtools_1.4.3     Biostrings_2.20.4   GenomicRanges_1.4.8
[4] IRanges_1.10.6

loaded via a namespace (and not attached):
[1] tools_2.13.1
```

C.4 Dependencies

The software `locsmoc` makes use of the following third-party features:

> `joptsimple 4.1` - A library for parsing command line options
> `http://pholser.github.com/jopt-simple/index.html`
>
> `colt 1.2.0` - a library for scientific and technical computing, used in fitting quadratic polynomials.
> `http://acs.lbl.gov/software/colt/`

References

[1] J Fridlyand, AM Snijders, D Pinkel, DG Albertson, and AN Jain. Hidden markov models approach to the analysis of array cgh data. J. Multivar. Anal., 90:132, 2004.

[2] P Hupe, N Stransky, JP Thiery, F Radvanyi, and E Barillot. Analysis of array cgh data: from signal ratio to gain and loss of dna regions. Bioinformatics, 20:3413–3422, 2004.

[3] H Li et al. The sequence alignment/map format and SAMtools. Bioinformatics, 25:2078–2079, 2009.

[4] The 1000 Genomes Project Consortium. A map of human genome variation from population-scale sequencing. Nature, 467:1061–1073, 2010.

[5] ES Venkatraman and AB Olshem. A faster circular binary segmentation algorithm for the analysis of array CGH data. Bioinformatics, 23(6):657–663, 2007.

[6] G Wallstrom, J Liebner, and RE Kaas. An implementation of bayesian adaptive regression splines (BARS) in C with S and R wrappers. Journal of Statistical Software, 26, 2008.

[7] H Willenbrock and J Fridlyand. A comparison study: applying segmentation to array cgh data for downstram analysis. Bioinformatics, 21(2):4084–4091, 2005.
